# Novel Variants in *LRRK2* and *GBA* Identified in Latino Parkinson Disease Cohort Enriched for Caribbean Origin

**DOI:** 10.3389/fneur.2020.573733

**Published:** 2020-11-12

**Authors:** Karen Nuytemans, Farid Rajabli, Parker L. Bussies, Katrina Celis, William K. Scott, Carlos Singer, Corneliu C. Luca, Angel Vinuela, Margaret A. Pericak-Vance, Jeff M. Vance

**Affiliations:** ^1^John P. Hussman Institute for Human Genomics, University of Miami Miller School of Medicine, Miami, FL, United States; ^2^Dr. John T. Macdonald Foundation Department of Human Genetics, University of Miami Miller School of Medicine, Miami, FL, United States; ^3^Division of Parkinson's Disease and Movement Disorders, Department of Neurology, University of Miami Miller School of Medicine, Miami, FL, United States; ^4^Movement Disorders Group, Manatí Medical Center, Neurosciences Institute, Manatí, Puerto Rico

**Keywords:** Parkinson disease, Hispanic/Latino, genetics, diversity and inclusion, health disparities

## Abstract

**Background:** The Latino population is greatly understudied in biomedical research, including genetics. Very little information is available on presence of known variants originally identified in non-Hispanic white patients or novel variants in the Latino population. The Latino population is admixed, with contributions of European, African, and Amerindian ancestries. Therefore, the ancestry surrounding a gene (local ancestry, LA) can be any of the three contributing ancestries and thus can determine the presence or risk effect of variants detected.

**Methods:** We sequenced the major exons and exons of reported Latino-specific variants in *GBA* and *LRRK2* and performed genome-wide genotyping for LA assessments in 79 Latino Parkinson disease (PD) patients, of which ~80% identified as Caribbean Latino.

**Results:** We observed five carriers of LRRK2 p.G2019S, one GBA p.T408M, and three GBA p.N409S on European as well as three GBA p.L13R on African LA backgrounds. Previous Latino variant GBA p.K237E was not observed in this dataset. A novel highly conserved and predicted damaging variant LRRK2 p.D734N was identified in two unrelated individuals with African LA. Additionally, we identified rare, functional variants LRRK2 p.P1480L and GBA p.S310G in one individual each heterozygous for European/Amerindian LA.

**Discussion:** Additional functional analysis will be needed to determine the pathogenicity of the novel variants in PD. However, the identification of novel disease variants in the Latino cohort potentially contributing to PD supports to importance of inclusion of Latinos in genetics research to provide insight in PD genetics in Latinos specifically as well as other populations with the same ancestral contributions.

## Introduction

Parkinson disease (PD) is the second most common neurodegenerative disease next to Alzheimer disease (AD), affecting individuals of all races and ethnicities. Most studies of PD, however, have been conducted in individuals of European (non-Hispanic whites, NHW) and Asian descent. Interestingly, incidence rates of PD are slightly higher in Latinos than for NHW (1, 2), indicating a clear disregard of the field to include Latinos in PD research. The bias toward NHW leads to health disparities for PD diagnosis and treatment. Many of the disparity reports however make no distinctions for NHW vs. Latinos, compared to for example NHW vs. African Americans (3). Therefore, the health disparities experienced by Latinos are likely understudied and underestimated despite the fact they are the fastest-growing and now largest minority in the US (18.3%) (4).

To date, >50 genes/loci have been identified for PD in European or Asian-descent studies (5). It is not known at what frequency NHW PD variants occur in other racial/ethnic groups or if entirely different variation or separate genes play a role in these other groups. Variants unique to a specific racial background have been reported for PD, such as *PINK1* variants that are predominantly identified in Asian patients (6). Ethnic-specific mutations have been found in several genes influencing complex disease, most notably in late-onset AD, and the effects of these genetic differences vary between populations (7–11).

Interestingly, genetic research in admixed populations such as the Latino population can provide insight in genetic contribution on many backgrounds because of their complex and variable genetic admixture. Latino populations collectively trace their ancestry to three continental groups; European, Amerindians, and West African (12–14), though contributions to contemporary Latino populations vary geographically (15–18). Interestingly, specifically for the Caribbean, there is high variability in ancestry contribution among and even within different Latino groups of this region (19). These contributions of various origins also lead to the observation that even though an individual's global ancestry (“average” ancestry) might mostly resemble European, African (American) or Amerindian, their genome is a mosaic of contributions. Therefore, local ancestry (LA), or the ancestral background of a particular (“local”) chromosomal region or haplotype (i.e., *LRRK2* locus), can be highly variable between different genomic regions and between individuals of the same population group. More recently, different variant size effects have been demonstrated for the same variant on different LA, i.e., lower risk of APOEε4 for AD on African vs. European or Japanese background (20), clearly indicating the importance of understanding LA for disease variants.

A small number of studies have reported results of genetic analyses in small (secondary) Latino datasets (21–26). These analyses often summarize across all Latino PD patients, regardless of ancestry, due to the small sample size. Given the high variability of admixture in these populations (described above), caution is warranted for the interpretation and extrapolation of these results. The only larger cohort, Latin American Research Consortium on the Genetics of PD (LARGE-PD, PI Dr. Mata) consisting of 1,150 Latino patients originating from southern South America, reports an enrichment of a novel variant in PD gene LRRK2 (p.Q1111H, rs78365431) in Peruvian and Chilean PD patients and controls (27) as well as a GBA mutation (p.K237E, rs773409311) in Colombian patients only (28), suggesting these variants originated from the Amerindian genetic background in these patients. Though these studies are an important first step, more elaborate analyses in the full range of Caribbean, Central, and South America are needed. The data presented here is the first report on variants in a cohort highly enriched for Caribbean Latino patients, complementing the reported dataset of LARGE-PD.

## Materials and Methods

### Human Subject Research Compliance

The presented study was approved by the Institutional Review Board at the University of Miami and informed consent for the survey was obtained from all participants.

### Sample Dataset

All PD participants were enrolled locally in Miami, FL, through collaboration with the University of Miami Department of Neurology Movement Disorders Division (Drs. Singer and Luca) or through ascertainment efforts in Puerto Rico through collaboration with Dr. Vinuela of the Movement Disorders Group at Manatí Medical Center in Manatí, PR.

### Genotyping Chip

We performed genome-wide genotyping using Illumina's Global Screen Assay (GSA) with Multiple Disease content version 2 (GSAMDv2), at the Center for Genome Technology at John P. Hussman Institute for Human Genomics. Quality control analyses were performed using the PLINK software, v.2 (29). Samples with a call rate <90% and with excess or insufficient heterozygosity (± 3 standard deviations) were excluded. Sex concordance was checked using X chromosome data. To eliminate duplicate and related samples, relatedness among the samples was estimated by using identity by descent (IBD). SNPs available in samples with the call rate <97%, or those not in Hardy–Weinberg equilibrium (*p* < 1x e-5), were eliminated from further analysis.

Illumina's CNVpartition program (Illumina, San Diego, CA) was used with default settings to evaluate presence of copy number variations in the genotyping data.

The genotyping data was used for determination of ancestries as well as presence of few variants (potentially) contributing to PD included on the chip (LRRK2 p.G2019S, p.Q1111H, and PARK2 p.R275W).

### Global and Local Ancestry Determination

Standard principal component analysis (PCA) using the Eigen-strat program (30) was performed to establish global ancestry for the participants. Reference datasets from the Human Genome Diversity Project (HGDP) data, i.e., European (/NHW), West African, Amerindian, were used in the analysis (31).

To determine LA at the genomic region surrounding the known PD genes, we phased the genotyping data using SHAPEITtoolver.2 (32) and the same reference datasets as for the PCA. We then used the RFMix ancestry software (33) to estimate LA for the whole genome (for reference) and around *LRRK2, GBA*, and *PARK2* in particular. These LA blocks are defined by variants common in specific ancestral populations spread across a large region, up to several Mb, depending on LD structure. The same reference populations (NHW, West African, Amerindian) used for phasing are used in the LA estimation. RFMix then compares each genomic region to the reference populations to infer the ancestral origin of each haplotype. Admixture plots identifying overall percentage of ancestral contributions are created using the ADMIXTURE program (34).

### Sanger Sequencing

We performed Sanger sequencing for exons in major PD genes for late-onset PD harboring known pathogenic variants (LRRK2 p.R1441 hotspot codon, p.G2019S, GBA common variants, SNCA), as well as harboring newly identified variants putatively contributing to Latino PD reported by Velez-Pardo et al. (28). Additionally, we extended LRRK2's analyses to all exons coding for functional domains Roc and Kinase, as well as exons harboring putative pathogenic variants identified in NHW patients in-house and by collaborators (personal communication). In total, these exons include LRRK2 exon 17-19, exon 29-31, exon 34, exon 36, exons 38-44, GBA exons 2-11, and SNCA exons 2-3 (primer sequences are available upon request).

### TaqMan Genotyping

To confirm the observed homozygous status of variant PARK2 p.R275W on the genotyping chip, we performed TaqMan genotyping (C__27532069_20, Thermo Fisher Scientific) on all participants using the recommended protocol. Data were analyzed on QuantStudio (Life Technologies).

### Variant Annotation

Novel variants are annotated for conservation (PhastCons/GERP, values over 2 and 0.5 are considered conserved by consensus) and functional effect in the protein using PolyPhen2 (35) as well as Combined Annotation Dependent Depletion algorithm (CADD) score. A score over 20 indicates top 1% of highest CADD scores (most evidence for functional potential of the position) genome-wide. Additionally, we queried the genome aggregation database [gnomAD, (36)] holding exonic/genomic data of 140,000 individuals, including 17,000 “Latino” individuals.

## Results

**Figure 1:**
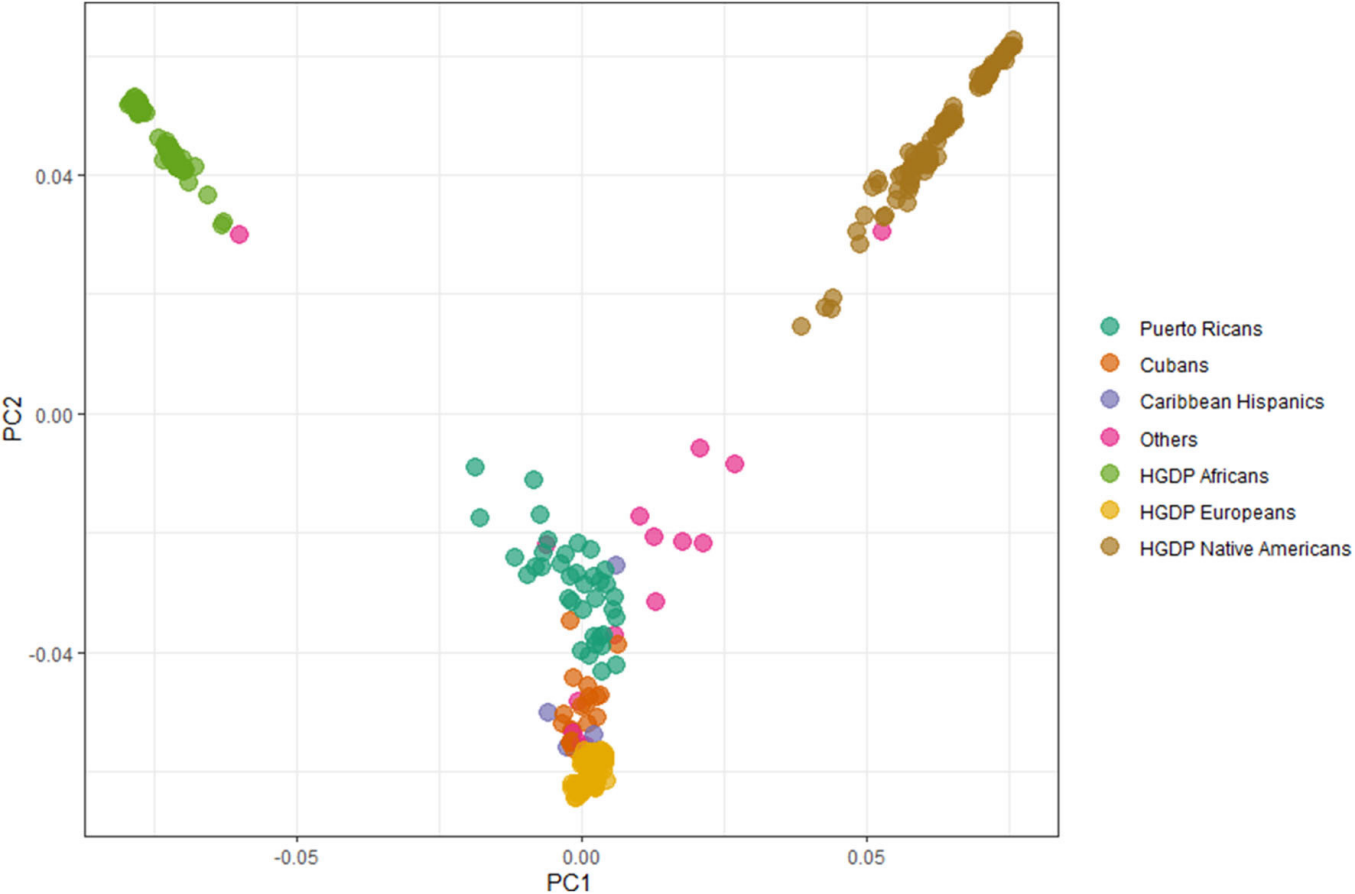
Principal component analyses. Estimation of relationship to ancestral groups from Human Genome Diversity Project (HGDP). Aqua = Puerto Ricans. Orange = Cubans. Purple = Caribbean Latinos (including Dominican Republic, mixed or undefined Caribbean origin). HGDP datasets included Europeans (yellow), Africans (green), and Amerindians (brown).

**Figure 2:**
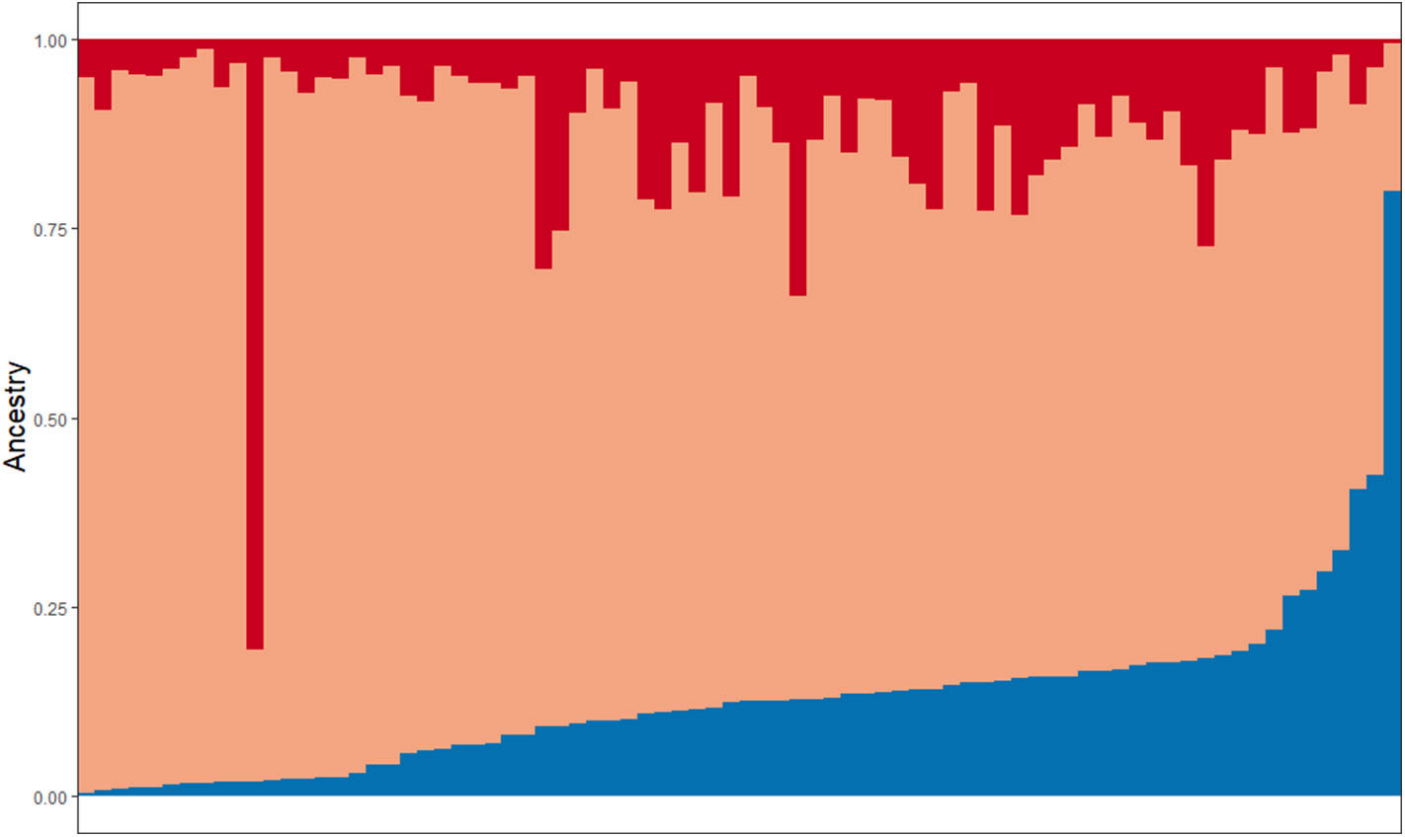
Representation of ancestral admixture in Latino cohort. Subjects are sorted on the X-axis based on percentage of Amerindian ancestry contribution (0–80%, displayed in blue). Colors in each vertical line represent that individual's ancestral admixture. Red = African, Orange = European, Blue = Amerindian.

A total of 79 Latino patients are included in this report, 79.7% identified as Caribbean (originating from Cuba, PR, Dominican Republic, or mixed/undefined). Other countries of origin reported by participants include Colombia, Peru, Ecuador, El Salvador, Guatemala, Brazil, Mexico, or unknown. Sample characteristics are described in Table 1. Nineteen out of 79 patients reported a first or second degree relative with PD (positive family history, FamHx+; 24%). Analyses of global ancestry (Figure 1) and ancestral contributions (admixtures, Figure 2) determined that the vast majority of this cohort has a high percentage of European ancestral contribution, though highly variable contribution from both African and Amerindian ancestry is observed (0 to ~80%, Figure 2). Contribution of other ancestries (e.g., East Asian) was minimal (<2%, data not shown).

**Table 1:** Sample characteristics.

	***N* (%)**	**Avg AAO (range)**	**M/F ratio**	**FamHx P/N+U**
ALL	79 (100)	54.4 (29–69)	42/37	19/60
Caribbean	63 (79.7)	63 (40–69)	36/27	16/47
Puerto rico	37 (46.8)	55.6 (40–67)	19/18	14/23
Cuba	22 (27.8)	53.6 (42–69)	15/7	1/21
Dominican republic	2 (2.5)	49 (1 unknown)	1/1	0/2
Undefined	2 (2.5)	57.5 (53–62)	1/1	1/1
Other	16 (20.3)	53.2 (29–68)	6/10	3/13
Colombia	2 (2.5)	55.5 (55–56)	0/2	0/2
Peru	2 (2.5)	54 (49–59)	0/2	1/1
El Salvador, Guatemala, Brazil, Ecuador, Mexico (1 each)	5 (6.3)	55.5 (44–68)	2/3	1/4
Unknown	7 (8.9)	50.2 (29–64)	4/3	1/6

*avg AAO, average age at onset; M/F, male/female; FamHx, family history (defined as first- or second-degree relative); P, positive; N, negative; U, unknown*.

### Detection of Known Variants in Selected Exons of Major PD Genes

We set out to determine the frequency of rare (MAF<1%) known variants originally identified in NHW patients in the Latino cohort. Using genotyping and Sanger sequencing data, we identified five heterozygous carriers of LRRK2 p.G2019S (5/79 = 6.3%, 1/19 FamHx+ = 5.3%) of various origins, two heterozygous carriers of GBA p.A495P (2/79 = 2.5%, 0/19 FanHx+) from Puerto Rico, three carriers of GBA p.N409S (3/79 = 3.8%, 0/19 FamHx+) of various origins, one heterozygous carrier of GBA p.T408M (1/79 = 1.2%, 0/19 FamHx+) from Cuba, three carriers of GBA p.L13R from Puerto Rico (3/79 = 3.8%, 1/19 FamHx+ = 5.3%), and a homozygous carrier of PARK2 p.R275W (confirmed by TaqMan genotyping, 1/79, 0/19 FamHx+ or 2/158 alleles = 1.2%) from Puerto Rico (Table 2). We did not observe any variants in *SNCA*, on the LRRK2 p.R1441 (C/G/H/S) hotspot or GBA p.L483P. No larger copy number variations in major PD genes detectable by the genotyping chip were observed.

**Table 2:** Known rare variants (MAF<1%) identified in major exons of PD genes.

**Gene**	**Variant**	**#carriers**	**Countries of origin**	**Avg AAO**	**Likely local ancestry**	**gnomAD overall (%)**
LRRK2	G2019S	5	2x PR, Brazil, Guatemala, unknown	56 (47–61)	European	0.05
GBA	T408M	1	Cuba	58	European	0.6
	N409S	3	Cuba, PR, unknown	55.7 (45–64)	European	0.2
	A495P	2	PR	Unknown	Amerindian/European	0.01
	L13R	3	PR	51.67 (41–59)	African	0.007

*MAF, minor allele frequency; Avg AAO, average age at onset; PR, Puerto Rico; gnomAD, genome aggregation database*.

We also evaluated presence of reported putative Latino specific and/or Latino PD contributing variants, i.e., LRRK2 p.Q1111H and GBA p.K237E. We did not observe either of these variants in the current dataset.

When examining the LA for *LRRK2, GBA*, and *PARK2* for the variant carriers, we determined that all variant carriers are homozygous for European LA at the genomic location where they carry a variant, except for one carrier of GBA p.A495P (Amerindian/European) and all three carriers of GBA p.L13R (2 × African/European and 1x African/Amerindian). Interestingly, p.L13R is common in the African population (7.7% in gnomAD), vs. <0.5% in other population groups, and considered benign for GBA function in ClinVar.

### Identification of Additional Variants in Selected Exons of Major PD Genes

We identified five heterozygous carriers of rare new variants (Table 3) with varying levels of *in-silico* support for pathogenicity (Table 4); LRRK2 p.D734N (2 individuals), p.P1480L, p.R1941H, and GBA p.S310G.

**Table 3:** New variants (MAF<1%) identified in selected exons of major PD genes.

**Gene**	**Variant**	***N***	**Country of origin**	**AAO**	**FamHx P/N**	**Likely local ancestry**	**gnomAD overall (%)**
LRRK2	D734N	2	PR	67 / 60	1/1	African	0.000008
	P1480L	1	Ecuador	44	0/1	Amerindian/European	Not observed
	R1941H	1	Cuba	42	0/1	European	0.000127
GBA	S310G	1	PR	58	0/1	Amerindian/European	0.000021

*MAF, minor allele frequency; AAO, age at onset; FamHx P/N, family history positive/negative; U, unknown; PR, Puerto Rico; gnomAD, genome aggregation database*.

**Table 4:** *In silico* evidence for novel variants.

**Gene**	**Variant**	**GERP**	**Phast Cons**	**PolyPhen2**	**CADD**	**gnomAD NFE (%)**	**gnomAD AFR (%)**	**gnomAD Lat (%)**	**gnomAD EAS (%)**
LRRK2	D734N	5.94	0.579	Probably damaging	25.5	0	0	0.00003	0
	P1480L	5.53	0.935	Probably damaging	29	–	–	–	–
	R1941H	4.85	1.000	Possibly damaging	23.6	0.00019	0.00004	0.00028	0.00005
GBA	S310G	3.51	0.985	benign	26.5	0	0.00004	0	0.00020

*gnomAD, genome aggregation database; NFE, non-Finish Europeans; AFR, African; Lat, Latinos; EAS, East Asian*.

The two individuals carrying the LRRK2 p.D734N variants are from PR; only one reports a positive family history. No DNA of the other affected in the family was available for segregation analyses. This variant has only been reported once in gnomAD in an additional Latino individual. LA analyses in these individuals (Amerindian/African and African/European) suggest that this variant might be located on an African background. The variant is predicted to be highly deleterious and is conserved. This variant has not been reported in PD context before, so no information is available in ClinVar. Both individuals presented with mild idiopathic PD with predominant tremor and postural changes and reported loss of smell and constipation. One further presented with short-term memory problems and the other with possible REM sleep behavior disorder.

The patient carrying LRRK2 p.P1480L variant identified Ecuador as country of origin and reported no positive family history to their knowledge. The variant was not present in 140,000 individuals from gnomAD (including 17,000 Latinos), though p.P1480S on the same codon is reported in only one European individual (0.000004% overall in gnomAD). The position is highly conserved, and the variant is predicted to be damaging. LA analyses showed that this patient is heterozygous for Amerindian and European ancestry at the *LRRK2* locus. The patient presented with idiopathic PD with tremor, bradykinesia, and rigidity and underwent successful deep brain stimulation surgery.

Variant LRRK2 p.R1941H was identified in a Cuban patient with no family history for PD, is classified as a Variant of Unknown Significance to PD in ClinVar, and has been observed in European, Latino, and African genomes in gnomAD (0.0001%). *In silico* predictions are inconsistent in supporting a damaging role of this variant. The individual carrying the variant is homozygous for European ancestry at the *LRRK2* locus. The patient presented with idiopathic PD with mild tremor, rigidity of the neck and leg, postural instability, and moderate facial hypokinesia.

The patient carrying GBA p.S310G is from PR and reports no family history for PD. The variant has been reviewed to be (likely) pathogenic for GBA function in ClinVar and has not been observed in European or Latino individuals from gnomAD but is rare in East Asian individuals. LA analyses identified both Amerindian and European ancestry at the *GBA* locus for the carrier. The patient presented with mild idiopathic PD with predominant tremor and postural changes.

## Discussion

Here we sequenced exons with reported pathogenic or strong risk variants for PD in three known PD genes (*LRRK2, GBA*, and *SNCA*) to evaluate presence of these variants originally identified in NHW patients in a Latino cohort enriched for Caribbean patients. Additionally, we extended LRRK2's analyses to all exons coding for functional domains Roc and Kinase, as well as exons harboring putative pathogenic variants identified in NHW patients in-house and by collaborators (personal communication). We used genome-wide genotyping data to determine ancestral background of identified variants and presence of few extra variants included on the chip (e.g., PARK2 R275W). We identified five carriers of LRRK2 p.G2019S as well as more common GBA p.T408M and p.N409S in one and three patients, respectively, all on putative European background. As these variants have been frequently reported in European patients, this suggests these variants were introduced to the Latino population through their European ancestor. Additionally, we identified benign variants GBA p.L13R common in African populations, in three individuals, and p.A495P in one individual. LA analyses supported p.L13R was indeed introduced through an African ancestor. p.A495P was identified in two patients who are heterozygous for European and Amerindian LA at the variant location. No ancestry-specific variants were located close enough (~10 kb) to the variant to allow us to phase the variants with its ancestral background (defined by variants across up to several Mb surrounding the gene) in cloning experiments. Independent of the reported observation here of GBA p.A495P, this variant has been identified across populations with rare instances reported in Africans, Latinos, East Asians, and Europeans in gnomAD. This reoccurrence on different backgrounds might indicate a tolerance of GBA for changes on this position, suggesting this variant is likely benign, which is also reflected in ClinVar's assessment of its relevance to GBA function.

Interestingly, we identified four more variants with varying levels of evidence for impact in PD. The presence of LRRK2 p.R1941H in individuals of all populations in gnomAD suggests tolerability for this variant, thus reducing the likelihood that this variant is a major player in PD. Data on LRRK2 p.D734N and p.P1480L and GBA p.S310G however support potential pathogenic roles of these variants. LRRK2 p.D734N is predicted to be highly functional and is very rare in the general population being identified only once in another Latino individual. Though one patient presented with positive family history, unfortunately no DNA was available for the others affected for segregation analyses. However, the observation of this variant in two independent PD patients on African LA, rarity in the general population (including African individuals), and strong *in silico* evidence supports the hypothesis that this variant might be a novel pathogenic variant for PD in individuals with African background. The identification in just Latino individuals, and not European or African groups, could suggest that this variant was introduced more recently in Latin history.

Both variants LRRK2 p.P1480L and GBA p.S310G have been identified each in one patient who is heterozygous for European and Amerindian LA at the variant location and does not report family history preventing segregation analyses. No ancestry-specific variants were located close enough (~10 kb) to either variant to allow for phasing of the variants on its ancestral background. Both variants are highly conserved and are predicted to have a (strong) effect on protein function. LRRK2 p.P1480L has not been reported previously in any general population, though a variant on the same codon (p.P1480S) was observed in one European individual. No information on this variant is available; however, it is located in the highly conformational Roc domain of LRRK2 and affects a proline residue, which are often involved in providing curvature in protein structures, suggesting a potential consequence for the domain structure due to this variant. Additional data of other carriers or families or functional analyses would be needed to assess its impact for PD. In contrast, GBA p.S310G has been observed in Gaucher's disease patients before and has been reviewed to be pathogenic by ClinVar. It has been seen very rarely in East Asian individuals in gnomAD. LA analyses in the variant carrier did not identify East Asian ancestry in this region (<1% in patient overall), indicating that this variant might have arisen independently in different populations. All patients carrying these new rare variants presented with classic idiopathic PD without atypical features; often with predominant tremor; and reporting no hallmarks differentiating them from other idiopathic PD. Screening in more (Caribbean) Latino PD cohorts or extensive single molecule sequencing will be needed in the future to confirm pathogenicity of these new potential PD variants and determine the ancestral origin of these variants in the Latino population. This first report on identification of novel variants in selected exons with higher likelihood of impactful variants in major PD genes in a Caribbean enriched cohort indicates that we can identify novel variants in the Latino population with variable evidence for involvement in PD pathogenesis. This is supported by the identification of the Colombian-specific variant GBA p.K237E (28) when querying GBA in a larger continental Latin dataset. Extending these analyses to more exons, more genes and larger cohorts will greatly increase the number of novel variants we identify in Latino PD patients and will further the field's understanding of PD in the Latino population.

Furthermore, inclusion of admixed population in genetic research is especially valuable because of their varied ancestry. As evidenced here by potential pathogenic variant LRRK2 p.D734N and previously by Velez-Pardo for GBA p.K237E, variants identified in Latino populations specifically can provide insight in variants on African and Amerindian background, both of which also play a major role in other, equally underserved, populations (African American/Amerindian).

Generally, the lack of information for other racial and ethnic populations (albeit in genetics specifically or biomedicine overall) leads to health disparities as study of a limited population pool creates biases in findings and only benefits the limited population in the end (37). Expanding genetic studies of complex diseases, such as PD, to Latino populations is crucial to meeting the needs of this increasing US demographic. The identification of novel variants in Latino cohorts not previously identified further support the importance of inclusion of participants across race/ethnicity.

## Data Availability Statement

All genotyping data will be available through dbGAP, accession number: phs000908.

## Ethics Statement

The studies involving human participants were reviewed and approved by Institutional Review Board at the University of Miami. The patients/participants provided their written informed consent to participate in this study.

## Author Contributions

KN, WS, MP-V, and JV contributed conception and design of the study. PB, KC, CS, CL, and AV were responsible for participant enrolment and data collection. FR performed the chip quality control and ancestry analyses. KN performed the sequencing analyses and wrote the first draft of the manuscript. WS, JV, CS, CL, and AV critically reviewed the manuscript. All authors contributed to manuscript revision and read and approved the submitted version.

## Conflict of Interest

The authors declare that the research was conducted in the absence of any commercial or financial relationships that could be construed as a potential conflict of interest.

## References

[1] Van Den Eeden SK Tanner CM Bernstein AL Fross RD Leimpeter A Bloch DA. Incidence of Parkinson's disease: variation by age, gender, and race/ethnicity. Am J Epidemiol. (2003) 157:1015–22. 10.1093/aje/kwg068 12777365

[2] Wright Willis A Evanoff BA Lian M Criswell SR Racette BA. Geographic and ethnic variation in Parkinson disease: a population-based study of US medicare beneficiaries. Neuroepidemiology. (2010) 34:143–51. 10.1159/000275491 20090375 PMC2865395

[3] Schneider MG Swearingen CJ Shulman LM Ye J Baumgarten M Tilley BC. Minority enrollment in Parkinson's disease clinical trials. Parkinsonism Relat Disord. (2009) 15:258–62. 10.1016/j.parkreldis.2008.06.005 18693062 PMC2700020

[4] U. S. Census Bureau Vintage 2018 Population Estimates Program. Washington, DC (2019).

[5] Bandres-Ciga S Diez-Fairen M Kim JJ Singleton AB. Genetics of Parkinson's disease: an introspection of its journey towards precision medicine. Neurobiol Dis. (2020) 137:104782. 10.1016/j.nbd.2020.104782 31991247 PMC7064061

[6] Nuytemans K Theuns J Cruts M Van Broeckhoven C. Genetic etiology of Parkinson disease associated with mutations in the SNCA, PARK2, PINK1, PARK7, and LRRK2 genes: a mutation update. Hum Mutat. (2010) 31:763–80. 10.1002/humu.21277 20506312 PMC3056147

[7] Farrer LA Cupples LA Haines JL Hyman B Kukull WA Mayeux R. Effects of age, sex, and ethnicity on the association between apolipoprotein E genotype and Alzheimer disease. a meta-analysis APOE and Alzheimer disease meta analysis consortium. JAMA. (1997) 278:1349–56. 9343467

[8] Collins FS. Shattuck lecture–medical and societal consequences of the human genome project. N Engl J Med. (1999) 341:28–37. 10.1056/NEJM199907013410106 10387940

[9] Calderon JL Baker RS Fabrega H Conde JG Hays RD Fleming E. An ethno-medical perspective on research participation: a qualitative pilot study. MedGenMed. (2006) 8:23. 16926762 PMC1785211

[10] Reitz C Jun G Naj A Rajbhandary R Vardarajan BN Wang LS. Variants in the ATP-binding cassette transporter (ABCA7), apolipoprotein E4,and the risk of late-onset Alzheimer disease in African Americans. JAMA. (2013) 309:1483–92. 10.1001/jama.2013.2973 23571587 PMC3667653

[11] Cukier HN Kunkle BW Vardarajan BN Rolati S Hamilton-Nelson KL Kohli MA. ABCA7 frameshift deletion associated with Alzheimer disease in African Americans. Neurol Genet. (2016) 2:e79. 10.1212/NXG.0000000000000079 27231719 PMC4871806

[12] Mao X Bigham AW Mei R Gutierrez G Weiss KM Brutsaert TD. A genomewide admixture mapping panel for hispanic/latino populations. Am J Hum Genet. (2007) 80:1171–8. 10.1086/518564 17503334 PMC1867104

[13] Price AL Patterson N Yu F Cox DR Waliszewska A McDonald GJ. A genomewide admixture map for latino populations. Am J Hum Genet. (2007) 80:1024–36. 10.1086/518313 17503322 PMC1867092

[14] Bryc K Auton A Nelson MR Oksenberg JR Hauser SL Williams S. Genome-wide patterns of population structure and admixture in West Africans and African Americans. Proc Natl Acad Sci USA. (2010) 107:786–91. 10.1073/pnas.0909559107 20080753 PMC2818934

[15] Guthery SL Salisbury BA Pungliya MS Stephens JC Bamshad M. The structure of common genetic variation in United States populations. Am J Hum Genet. (2007) 81:1221–31. 10.1086/522239 17999361 PMC2276358

[16] Wang S Ray N Rojas W Parra MV Bedoya G Gallo C. Geographic patterns of genome admixture in Latin American Mestizos. PLoS Genet. (2008) 4:e1000037. 10.1371/journal.pgen.1000037 18369456 PMC2265669

[17] Choudhry S Coyle NE Tang H Salari K Lind D Clark SL. Population stratification confounds genetic association studies among latinos. Hum Genet. (2006) 118:652–64. 10.1007/s00439-005-0071-3 16283388

[18] Silva-Zolezzi I Hidalgo-Miranda A Estrada-Gil J Fernandez-Lopez JC Uribe-Figueroa L Contreras A. Analysis of genomic diversity in Mexican mestizo populations to develop genomic medicine in Mexico. Proc Natl Acad Sci USA. (2009) 106:8611–6. 10.1073/pnas.0903045106 19433783 PMC2680428

[19] Moreno-Estrada A Gravel S Zakharia F McCauley JL Byrnes JK Gignoux CR. Reconstructing the population genetic history of the Caribbean. PLoS Genet. (2013) 9:e1003925. 10.1371/journal.pgen.1003925 24244192 PMC3828151

[20] Rajabli F Feliciano BE Celis K Hamilton-Nelson KL Whitehead PL Adams LD. Ancestral origin of ApoE epsilon4 Alzheimer disease risk in Puerto Rican and African American populations. PLoS Genet. (2018) 14:e1007791. 10.1371/journal.pgen.1007791 30517106 PMC6281216

[21] Saunders-Pullman R Cabassa J San Luciano M Stanley K Raymond D Ozelius LJ. LRRK2 G2019S mutations may be increased in Puerto Ricans. Mov Disord. (2011) 26:1772–3. 10.1002/mds.23632 21449009 PMC3140626

[22] Alcalay RN Caccappolo E Mejia-Santana H Tang MX Rosado L Ross BM. Frequency of known mutations in early-onset Parkinson disease: implication for genetic counseling: the consortium on risk for early onset Parkinson disease study. Arch Neurol. (2010) 67:1116–22. 10.1001/archneurol.2010.194 20837857 PMC3329730

[23] Marder KS Tang MX Mejia-Santana H Rosado L Louis ED Comella CL. Predictors of parkin mutations in early-onset Parkinson disease: the consortium on risk for early-onset Parkinson disease study. Arch Neurol. (2010) 67:731–8. 20558392 10.1001/archneurol.2010.95 PMC3329757

[24] Deng H Le W Guo Y Hunter CB Xie W Huang M. Genetic analysis of LRRK2 mutations in patients with Parkinson disease. J Neurol Sci. (2006) 251:102–6. 10.1016/j.jns.2006.09.017 17097110

[25] Duque AF Lopez JC Benitez B Hernandez H Yunis JJ Fernandez W. Analysis of the LRRK2 p.G2019S mutation in Colombian Parkinson's disease patients. Colomb Med. (2015) 46:117–21. 10.25100/cm.v46i3.1553 26600626 PMC4640433

[26] Gatto EM Parisi V Converso DP Poderoso JJ Carreras MC Marti-Masso JF. The LRRK2 G2019S mutation in a series of Argentinean patients with Parkinson's disease: clinical and demographic characteristics. Neurosci Lett. (2013) 537:1–5. 10.1016/j.neulet.2013.01.011 23340200

[27] Mata IF Wilhoite GJ Yearout D Bacon JA Cornejo-Olivas M Mazzetti P. Lrrk2 p.Q1111H substitution and Parkinson's disease in Latin America. Parkinsonism Relat Disord. (2011) 17:629–31. 10.1016/j.parkreldis.2011.05.003 21632271 PMC3167927

[28] Velez-Pardo C Lorenzo-Betancor O Jimenez-Del-Rio M Moreno S Lopera F Cornejo-Olivas M. The distribution and risk effect of GBA variants in a large cohort of PD patients from Colombia and Peru. Parkinsonism Relat Disord. (2019) 63:204–8. 10.1016/j.parkreldis.2019.01.030 30765263 PMC7175776

[29] Purcell S Neale B Todd-Brown K Thomas L Ferreira MA Bender D. PLINK: a tool set for whole-genome association and population-based linkage analyses. Am J Hum Genet. (2007) 81:559–75. 10.1086/519795 17701901 PMC1950838

[30] Price AL Patterson NJ Plenge RM Weinblatt ME Shadick NA Reich D. Principal components analysis corrects for stratification in genome-wide association studies. Nat Genet. (2006) 38:904–9. 10.1038/ng1847 16862161

[31] Cann HM. Human genome diversity. C R Acad Sci III. (1998) 321:443–6. 10.1016/S0764-4469(98)80774-9 9769857

[32] Delaneau O Marchini J 1000 Genomes Project Consortium, 1000 Genomes Project Consortium. Integrating sequence and array data to create an improved 1000 *genomes project haplotype reference panel*. Nat Commun. (2014) 5:3934. 10.1038/ncomms4934 25653097 PMC4338501

[33] Maples BK Gravel S Kenny EE Bustamante CD. RFMix: a discriminative modeling approach for rapid and robust local-ancestry inference. Am J Hum Genet. (2013) 93:278–88. 10.1016/j.ajhg.2013.06.020 23910464 PMC3738819

[34] Alexander DH Novembre J Lange K. Fast model-based estimation of ancestry in unrelated individuals. Genome Res. (2009) 19:1655–64. 10.1101/gr.094052.109 19648217 PMC2752134

[35] Adzhubei IA Schmidt S Peshkin L Ramensky VE Gerasimova A Bork P. A method and server for predicting damaging missense mutations. Nat Methods. (2010) 7:248–9. 10.1038/nmeth0410-248 20354512 PMC2855889

[36] Karczewski KJ Francioli LC Tiao G Cummings BB Alfoldi J Wang Q. The mutational constraint spectrum quantified from variation in 141,456 humans. Nature. (2020) 581:434–43. 10.1038/s41586-020-2308-7 32461654 PMC7334197

[37] Bustamante CD Burchard EG De la Vega FM. Genomics for the world. Nature. (2011) 475:163–5. 10.1038/475163a 21753830 PMC3708540

